# Elevated Pro-Inflammatory Cell-Free MicroRNA Levels in Cerebrospinal Fluid of Premature Infants after Intraventricular Hemorrhage

**DOI:** 10.3390/ijms21186870

**Published:** 2020-09-19

**Authors:** Zsolt Fejes, Judit Erdei, Marianna Pócsi, Jun Takai, Viktória Jeney, Andrea Nagy, Alíz Varga, Attila Bácsi, László Bognár, László Novák, János Kappelmayer, Béla Nagy

**Affiliations:** 1 Department of Laboratory Medicine, Faculty of Medicine, University of Debrecen, H-4032 Debrecen, Hungary; fejes.zsolt@med.unideb.hu (Z.F.); pocsi.marianna@med.unideb.hu (M.P.); jun_takai@hotmail.com (J.T.); kappelmayer@med.unideb.hu (J.K.); 2 Kálmán Laki Doctoral School of Biomedical and Clinical Sciences, Faculty of Medicine, University of Debrecen, H-4032 Debrecen, Hungary; 3 MTA-DE Lendület Vascular Pathophysiology Research Group, Research Centre for Molecular Medicine, Faculty of Medicine, University of Debrecen, H-4032 Debrecen, Hungary; jutka.erdei@gmail.com (J.E.); jeneyv@belklinika.com (V.J.); 4 Doctoral School of Molecular Cell and Immune Biology, Faculty of Medicine, University of Debrecen, H-4032 Debrecen, Hungary; 5 Department of Pediatrics, Faculty of Medicine, University of Debrecen, H-4032 Debrecen, Hungary; nagyand@med.unideb.hu; 6 Department of Immunology, Faculty of Medicine, University of Debrecen, H-4032 Debrecen, Hungary; varga.aliz@med.unideb.hu (A.V.); etele@med.unideb.hu (A.B.); 7 Department of Neurosurgery, Faculty of Medicine, University of Debrecen, H-4032 Debrecen, Hungary; bognar.laszlo@med.unideb.hu (L.B.); lnovak@med.unideb.hu (L.N.)

**Keywords:** cerebrospinal fluid, intraventricular hemorrhage, inflammation, microRNA, oxidized hemoglobin, heme

## Abstract

Intraventricular hemorrhage (IVH) represents a high risk of neonatal mortality and later neurodevelopmental impairment in prematurity. IVH is accompanied with inflammation, hemolysis, and extracellular hemoglobin (Hb) oxidation. However, microRNA (miRNA) expression in cerebrospinal fluid (CSF) of preterm infants with IVH has been unknown. Therefore, in the present study, candidate pro-inflammatory cell-free miRNAs were analyzed in CSF samples from 47 preterm infants with grade III or IV IVH vs. clinical controls (*n* = 14). miRNAs were quantified by RT-qPCR, normalized to “spike-in” cel-miR-39. Oxidized Hb and total heme levels were determined by spectrophotometry as well as IL-8, VCAM-1, ICAM-1, and E-selectin concentrations by ELISA. To reveal the origin of the investigated miRNAs, controlled hemolysis experiments were performed in vitro; in addition, human choroid plexus epithelial cell (HCPEpiC) cultures were treated with metHb, ferrylHb, heme, or TNF-α to replicate IVH-triggered cellular conditions. Levels of miR-223, miR-155, miR-181b, and miR-126 as well as Hb metabolites along with IL-8 were elevated in CSF after the onset of IVH vs. controls. Significant correlations were observed among the miRNAs, oxidized Hb forms, and the soluble adhesion molecules. During the post-IVH follow-up, attenuated expression of miRNAs and protein biomarkers in CSF was observed upon elimination of Hb metabolites. These miRNAs remained unaffected by a series of artificially induced hemolysis, which excluded red blood cells as their origin, while stimulation of HCPEpiCs with oxidized Hb fractions and heme resulted in increased extracellular miRNA levels in the cell culture supernatant. Overall, the hemorrhage-induced CSF miRNAs reflected inflammatory conditions as potential biomarkers in preterm IVH.

## 1. Introduction

Incidence of intraventricular hemorrhage (IVH) in premature infants has not changed substantially in the last two decades despite recent clinical improvements in diagnostic and surgical interventions [1,2]. IVH still affects about 15–20% of infants born preterm with very low birth weight [3], and prolonged consequences of the disease represent one of the leading causes of morbidity and mortality [2]. Due to immature blood vessels of germinal matrix, periventricular regions are increasingly vulnerable to spontaneous bleeding due to the rupture of vasculature [2]. Severity of IVH expands from mild to severe forms (grades I–IV); the original classification describes IVH with ventricular dilation as grade III and IVH with concomitant intraparenchymal hemorrhage as grade IV [4]. These conditions often result in impaired neurodevelopment, cerebral palsy, and mental retardation in surviving infants [2].

In case of severe IVH, extravasated blood accumulates in the intraventricular space, causing release of extracellular hemoglobin (Hb) into the cerebrospinal fluid (CSF) [5]. Cell-free Hb, in turn, undergoes an oxidative modification, forming oxidized metabolites, such as methemoglobin (metHb, Fe^3+^) and ferrylhemoglobin (ferrylHb, Fe^4+^) with a subsequent release of heme [6]. These oxidized Hb forms and heme exert various cytotoxic and pro-inflammatory effects after intracerebral and intraventricular hemorrhage, such as neuroinflammation with dysfunction of choroid plexus epithelial cells and brain microvascular endothelial cells (EC) via induction of the nuclear factor kappa-B (NF-κB) pathway [7,8,9]. Heme induces Toll-like receptor 4 (TLR4) activation with lipid peroxidation and programmed cell death of different cell types [10]. As a result, pro-inflammatory cytokines such as interleukin-8 (IL-8), tumor necrosis factor α (TNF-α), and IL-1β are produced at large quantity [11]. Furthermore, in extremely preterm infants increased serum IL-6 levels were associated with decreased activity of coagulation factor VII (FVII) that was found to be an independent risk factor for the development of IVH [12]. Inflamed choroid plexus epithelium adhesion receptors, such as intercellular adhesion molecule-1 (ICAM-1), have been found to be upregulated [13], which regulates trafficking of immune cells with the help of P-selectin into the cerebral ventricles [14]. Clinically, the importance of this inflammatory mechanism has been further supported by previous observations of elevated ICAM-1, vascular adhesion molecule-1 (VCAM-1), and E-selectin levels in CSF in patients with subarachnoid hemorrhage (SAH) [15] and bacterial sepsis with meningitis [16].

MicroRNAs (miRNAs) are small noncoding RNAs that contribute to posttranscriptional regulation of various gene expression via interfering with translation and promoting messenger RNA degradation, leading to attenuation of target protein expression [17]. Among numerous functions, they have been demonstrated to regulate various EC activation-dependent cellular events via the NF-κB signaling [18]. For instance, ICAM-1 exposure on EC is regulated by miR-223 [19], elevated miR-155 induces the production of pro-inflammatory cytokines in the vasculature [20], and VCAM-1 is regulated by both miR-126 [21] and miR-181b [22,23], while E-selectin expression is also modulated by the latter miRNA [22,23]. The role of miRNAs has been intensively studied in central nervous system (CNS) development and different neurological disorders, as well reviewed previously [24]. Upon brain injury, miRNAs are released from damaged tissue and cerebral vasculature into the subarachnoid space [25]. Increased CSF miRNA levels have been linked to the development of delayed cerebral ischemia (DCI) in adults with SAH [26]. However, to date, the expression of cell-free miRNAs in CSF has not been investigated in preterm infants after IVH.

In this study, we hypothesized that the levels of extracellular miRNAs were increased in CSF samples of premature infants upon the onset of IVH and reflected the inflammatory conditions induced by Hb oxidation products. Additionally, these CSF miRNAs were supposed to correlate with different pro-inflammatory protein biomarkers and the degree of choroid plexus epithelial cell activation. To replicate IVH-induced cellular disorders, human choroid plexus epithelial cells (HCPEpiC) were treated in vitro with oxidative Hb metabolites, heme, or recombinant TNF-α as control to analyze the alteration of secreted miRNAs. The origin of miRNAs was further studied using in vitro controlled hemolysis experiments.

## 2. Results

### 2.1. Baseline Characteristics of Study Participants

Forty-seven preterm infants with IVH and 14 age- and gender-matched clinical controls were enrolled into this study. Except for serum procalcitonin (PCT), no significant differences were found in blood and serum parameters between the two cohorts. In contrast, red blood cell (RBC) and white blood cell (WBC) counts as well as CNS injury-sensitive biomarkers in CSF (i.e., total protein, S100B, and lactate) were significantly elevated (*p* < 0.001) in IVH patients vs. controls during routine laboratory analyses (Table 1). Recruited subjects were premature infants diagnosed with grade III or IV IVH based on ultrasound findings. These patients were further subclassified to determine if the severity of IVH influenced the levels of CSF parameters. WBC count (data not shown) and RBC count, S100B, total protein with lactate concentrations in CSF, were significantly higher in both grade III and grade IV IVH groups compared to controls; however, there were no differences in these laboratory parameters between the two IVH severity subcohorts (Supplementary Figure S1A–D).

### 2.2. Elevated miRNA Levels in CSF after the Onset of Preterm IVH

**Figure 1:**
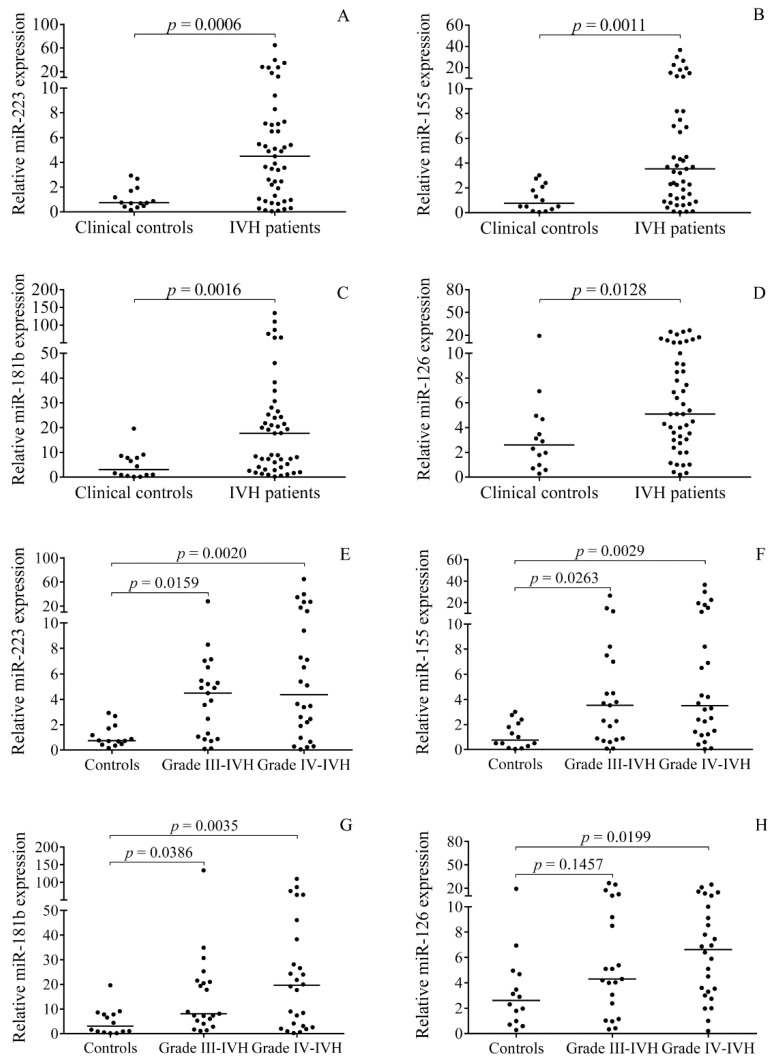
Analysis of CSF miRNA levels after the onset of IVH. Relative expressions of miR-223 (**A**), miR-155 (**B**), miR-181b (**C**), and miR-126 (**D**) were significantly higher in the entire IVH group (*n* = 47) versus controls (*n* = 14). However, no differences in miRNAs were seen between grades III (*n* = 21) and grade IV (*n* = 26) IVH (**E**–**H**). Dots represent single expression values. Median values are depicted. To compare the data of the two groups, Mann–Whitney U test was applied (**A**–**D**). Kruskal–Wallis test with Dunn’s multiple comparisons test was performed for the comparison of three groups (**E**–**H**). CSF: Cerebrospinal fluid, IVH: Intraventricular hemorrhage, miRNA: MicroRNA.

First, the expression of selected pro-inflammatory cell-free miRNAs in CSF after the onset of premature IVH was investigated, and the relative expression of miR-223 (*p* = 0.0006), miR-155 (*p* = 0.0011), miR-181b (*p* = 0.0016), and miR-126 (*p* = 0.0128) was found to be significantly higher in the IVH group vs. controls (Figure 1A–D). Although these CSF miRNAs showed a large variation in IVH, interestingly, the impact of disease severity on miRNAs was not observed between grades III and IV. Of note, increased expressions of miR-223, miR-155, and miR-181b were found in both subgroups (*p* < 0.05), while miR-126 showed a significant elevation (*p* = 0.0199) only in patients with grade IV IVH (Figure 1E–H). These results suggest that certain miRNAs in CSF are abnormally expressed after the onset of IVH.

### 2.3. Time-Dependent Alteration in miRNAs in Post-IVH CSF Samples

**Figure 2:**
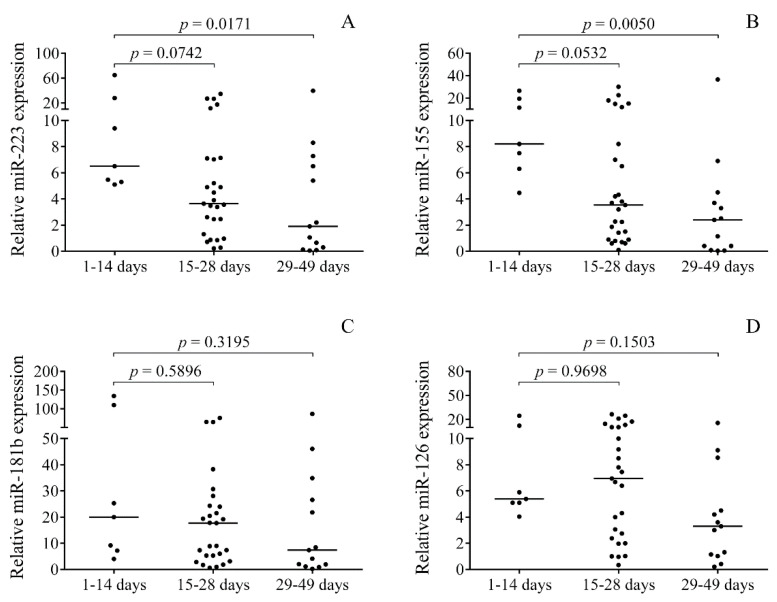
CSF samples were obtained at different time points after the onset of IVH; accordingly, the samples were subdivided into three groups, 1–14 days (*n* = 7), 15–28 days (*n* = 27), and 29–49 days (*n* = 13). Even higher miR-223 (**A**) and miR-155 (**B**) expressions were detected in those specimens collected within two weeks after the onset of IVH compared to those collected afterwards. However, miR-181b (**C**) and miR-126 (**D**) did not significantly alter within this time period. Dots represent single expression values. Median values are depicted. Kruskal–Wallis test with Dunn’s multiple comparisons test was performed for the comparison. CSF: Cerebrospinal fluid, IVH: Intraventricular hemorrhage.

Since baseline CSF samples were originally collected for diagnostic purposes, specimens were efficiently obtained in an organized manner at different time points after the onset of IVH (1–49 days, 23.8 ± 10.7 days) for further analysis. Based on the elapsed time between the onset of IVH and CSF sampling, the samples were categorized into three subgroups to analyze miRNAs over time: 0–14 days, 15–28 days, and 29–49 days (Figure 2). Significantly higher CSF miR-223 (*p* = 0.0171) and miR-155 (*p* = 0.0050) levels were found at early time points, i.e., 1–14 days after IVH, compared to those who had samples only after four weeks from the onset (Figure 2A,B). Conversely, miR-181b and miR-126 demonstrated no substantial changes within this time frame (Figure 2C,D). Based on these data, changes in CSF miRNA expression may be dependent on time elapsed from the onset of IVH.

### 2.4. Relationship between the Levels of Oxidized Hb Forms and miRNAs in CSF after IVH

**Figure 3:**
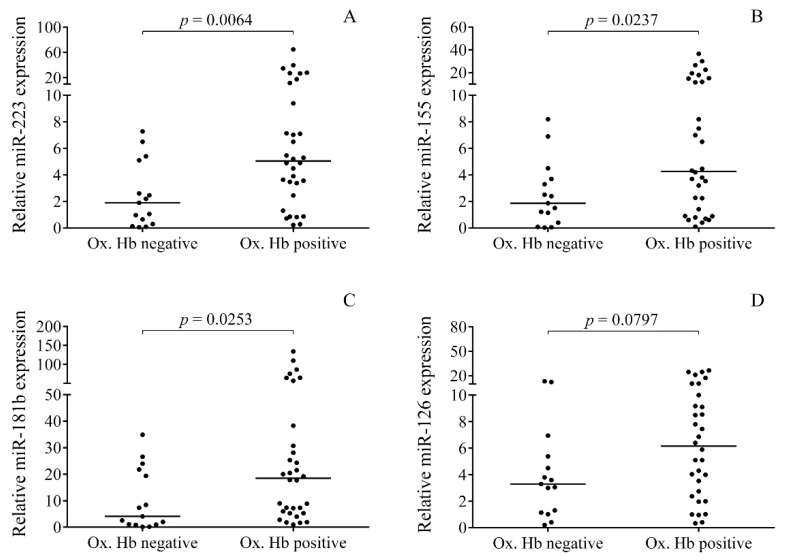
Oxidized forms of Hb were detected in 68% (*n* = 32) of all baseline CSF samples. Thus, IVH samples were further subclassified based on the status of oxidized Hb content to investigate how the presence of oxidized Hb influenced cell-free miRNA levels. Significant upregulation was revealed for CSF miR-223 (**A**), miR-155 (**B**), and miR-181b (**C**) in oxidized Hb positive vs. negative subjects (*n* = 15), while oxidized Hb content did not affect miR-126 levels (**D**). Dots represent single expression values. Median values are depicted. Mann–Whitney U test was performed for the comparison. CSF: Cerebrospinal fluid, IVH: Intraventricular hemorrhage, miRNA: MicroRNA, Ox. Hb: Oxidized hemoglobin.

Oxidized Hb forms, i.e., metHb (mean ± SD, 33.05 ± 50.73 μmol/L) and ferrylHb (3.93 ± 23.86 μmol/L), were detected in 68% of CSF samples (*n* = 32) after the onset of IVH in premature patients (data not shown). In addition, heme was detected in almost all the IVH samples (*n* = 45) with a mean concentration of 226.3 ± 262.9 μmol/L (Table 1). Non-IVH control samples showed no ferrylHb content with low level of metHb (1.82 ± 5.84 μmol/L) and heme (0.78 ± 2.81 μmol/L) (Table 1). Our group recently demonstrated that Hb-derived metabolites play a crucial role in the induction of pro-inflammatory response after IVH [27]. To investigate the association of the absence or presence of oxidized Hb forms with CSF miRNA expression, IVH samples were split into two subcohorts. Significantly higher miR-223 (*p* = 0.0064), miR-155 (*p* = 0.0237), and miR-181b (*p* = 0.0253) were observed in oxidized Hb positive vs. negative samples (Figure 3A–C). In case of miR-126, no significant relationship was found with oxidized Hb content (*p* = 0.0797) (Figure 3D). These data support that CSF miRNA levels are more induced in those premature IVH cases in which oxidized Hb metabolites accumulated due to massive hemolysis and extracellular Hb oxidation.

**Figure 4:**
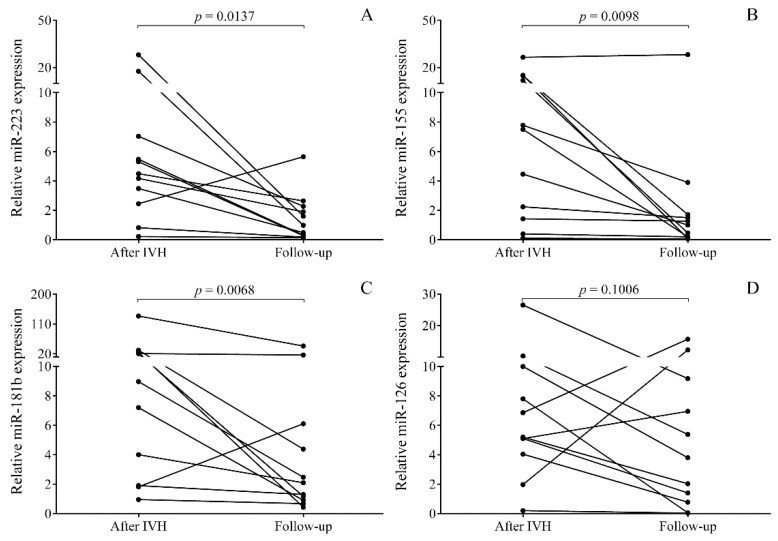
In those IVH patients (*n* = 11) with whom follow-up specimens were available, a positive correlation was observed between the change in oxidized Hb forms and miRNAs over time. In these patients, oxidized Hb content was not detectable by the end of follow-up period. IVH-induced elevation of miR-223, miR-155, and miR-181b was diminished (**A**–**C**) during the follow-up phase, while miR-126 did not show significant alteration (**D**). Dots represent single expression values of the pairs. Wilcoxon matched-pairs sign rank test or paired t-test was performed for the comparison. Hb: Hemoglobin, IVH: Intraventricular hemorrhage, miRNA: MicroRNA.

Next, our attention was drawn to determine if miRNAs in CSF were able to reflect the alteration of oxidized Hb content over time during the follow-up of preterm IVH. Additional CSF specimens were obtained from 18 IVH subjects with a total elapsed time of 85.4 ± 14.6 days. The following outcomes were typically seen in this study in terms of the change in oxidized Hb levels between baseline and follow-up samples: (1) High baseline oxidized Hb content significantly reduced and became undetectable (22.3 ± 19.1 vs. 0 μmol/L, *n* = 11) in the follow-up samples (Figure 4A–D), (2) remained oxidized Hb positive (9.27 ± 5.4 vs. 24.3 ± 18.9 μmol/L, *n* = 2) during the follow-up, and (3) stayed oxidized Hb negative (*n* = 5) throughout the study (data not shown). In those 11 oxidized Hb positive IVH individuals who actually showed resolution of IVH under therapy, the expression of miR-223, miR-155, and miR-181b was significantly reduced in the follow-up samples, containing no oxidized Hb (*p* < 0.05) (Figure 4A–C). In contrast, miR-126 did not show a substantial reduction in the same specimens (Figure 4D). On the other hand, sustainedly increased miRNA expression was revealed in samples showing constant positivity for oxidized Hb forms under treatment (data not shown). These results imply that miR-223, miR-155, and miR-181b expressions are highly associated with the production of pro-inflammatory oxidized Hb forms in CSF and can monitor IVH resolution with the improvement of clinical condition under treatment.

### 2.5. Correlations between Pro-Inflammatory Protein Biomarkers and miRNAs in Post-IVH CSF Samples

**Figure 5:**
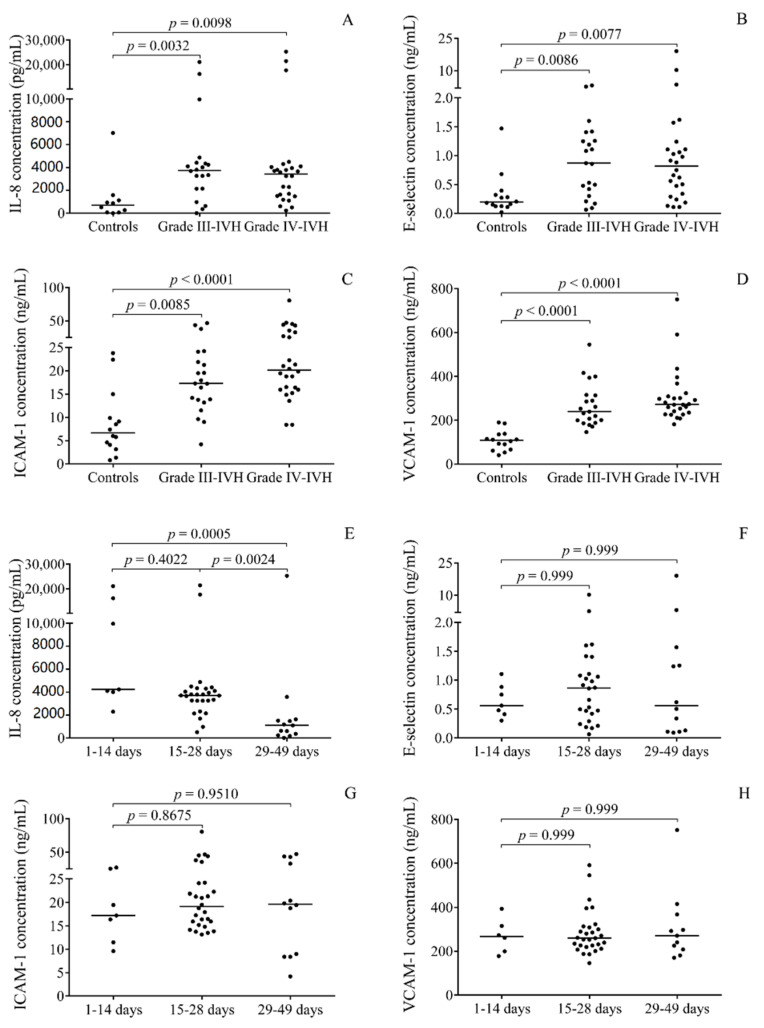
Augmented soluble IL-8 (**A**), E-selectin (**B**), ICAM-1 (**C**), and VCAM-1 (**D**) protein concentrations were detected by ELISA in baseline CSF samples of grade III (*n* = 21) and grade IV (*n* = 26) IVH vs. controls (*n* = 14). Since CSF samples were obtained at different time points after the onset of IVH, the samples were subdivided into three groups: 1–14 days (*n* = 7), 15–28 days (*n* = 27), and 29–49 days (*n* = 13). Only IL-8 (**E**) was significantly low at post-IVH days 29–49 and soluble adhesion molecules showed no change (**F**–**H**). Dots represent single values. Median values are depicted. Kruskal–Wallis test with Dunn’s multiple comparisons test or ANOVA with Bonferroni’s multiple comparisons test was performed for the comparisons. CSF: Cerebrospinal fluid, ICAM-1: Intercellular adhesion molecule 1, IL-8: Interleukin-8, IVH: Intraventricular hemorrhage, VCAM-1: Vascular cell adhesion molecule 1.

It has recently been reported that concentrations of pro-inflammatory markers, such as cytokines and chemokines (e.g., IL-8) and soluble adhesion molecules (e.g., VCAM-1), were upregulated in post-IVH CSF samples [27,28]. To address this question regarding altered miRNA expression in the present study setting, the levels of IL-8 with soluble E-selectin, ICAM-1, and VCAM-1 were measured by enzyme-linked immunosorbent assays (ELISA) in the post-IVH CSF samples. Their concentrations were first analyzed in relation to IVH severity and time course in the baseline CSF samples. As shown in Figure 5, these biomarkers showed significantly elevated concentrations in both grades III and IV IVH compared to controls, while—similar to miRNAs—no difference was observed between the two subgroups based on disease severity (Figure 5A–D). When time dependent alterations of these biomarkers were further studied using the same subgroups as above, only IL-8 was significantly lower at post-IVH days 29–49 and soluble adhesion molecules showed no change (Figure 5E–H). These findings indicate the activation of multiple cell types such as endothelial/epithelial cells in the choroid plexus as well as leukocytes occurred after the onset of IVH, while enhanced inflammatory response was lowered at later time points. Notably, reduced E-selectin, ICAM-1, VCAM-1, and IL-8 concentrations were analyzed under treatment with a total elapsed time of 85.4 ± 14.6 days in comparison to baseline samples (Supplementary Figure S2A–D).

Next, the relationships between protein biomarkers and miRNA expression in CSF were systematically characterized using Spearman’s test. Based on these statistical analyses, all of the four miRNAs were positively correlated with RBC count, oxidized Hb, and total heme levels as well as E-selectin concentrations. Positive correlations of miR-223, miR-155, and miR-181b were also shown with WBC count, IL-8, ICAM-1, and VCAM-1 (Table 2). These data underline the strong association of these miRNA levels with the production of oxidized Hb forms and dysfunction of choroid plexus epithelium in premature IVH.

### 2.6. Impact of Hemolysis on miRNAs Based on In Vitro Controlled Hemolysis Experiments

**Figure 6:**
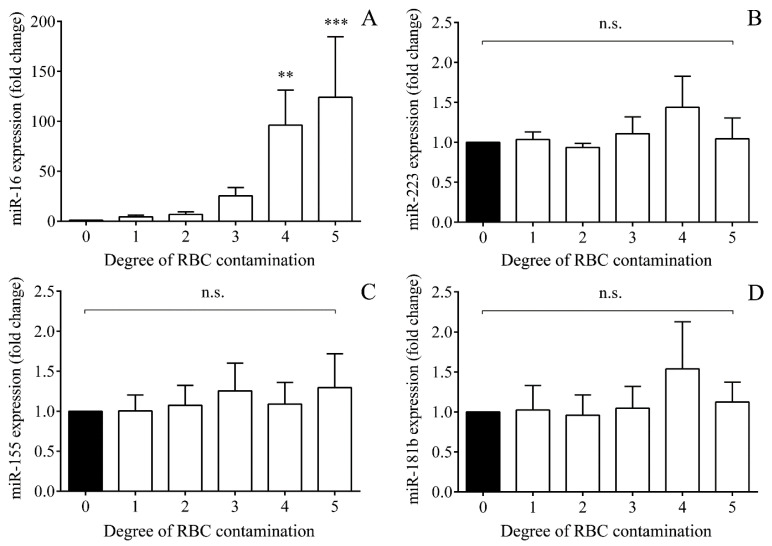
In vitro controlled hemolysis experiments (*n* = 5/condition) were performed to investigate whether these miRNAs were released from RBCs upon IVH. Hemolysis was artificially produced in plasma samples by adding RBCs in different degree (0, 1, 2.5, 5, 10, and 50% v/v). Artificial hemolysis at different extents led to the expression of RBC-specific miR-16 in the same tendency (**A**); however, even a massive hemolysis did not influence miR-223 (**B**), miR-155 (**C**), or miR-181b (**D**) levels. Mean ± SEM are depicted. ** *p* < 0.010, *** *p* < 0.001, n.s.: Not significant based on Friedman test with Dunn’s multiple comparisons test or ANOVA with Bonferroni’s multiple comparisons test. IVH: Intraventricular hemorrhage, miRNA: MicroRNA, RBC: Red blood cell.

In vitro controlled hemolysis experiments were performed to investigate whether these miRNAs with elevated levels were released from RBCs upon premature IVH. Hemolysis was artificially mimicked in plasma samples in vitro by adding isolated RBCs starting from 1 to 50% v/v including uncontaminated plasma sample (0% *v/v*) (Supplementary Figure S3A,B). miRNA levels in hemolytic and nonhemolytic control samples were quantified by RT-qPCR. As a positive control miRNA, RBC-specific miR-16 [29,30] was used to evaluate the degree of induced hemolysis. Gradually elevated miR-16 expression was observed, corresponding to degree of RBC contamination (Figure 6A); on the other hand, miR-223, miR-155, and miR-181b levels remained unaffected (Figure 6B–D). Accordingly, it is suggestive enough of RBC not being an origin of studied pro-inflammatory miRNAs due to in vivo hemolysis after the onset of IVH.

### 2.7. miRNA Levels in the Supernatant of Human Choroid Plexus Epithelial Cell Cultures after Treatment with Oxidized Hb Forms and Heme

**Figure 7:**
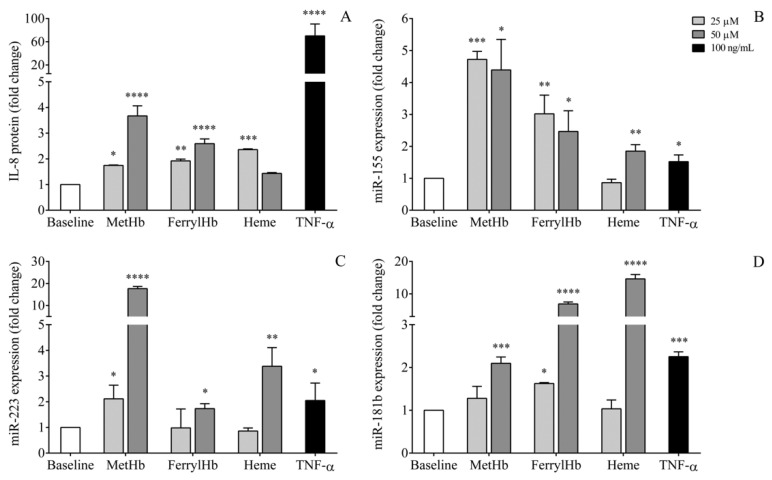
Human choroid plexus epithelial cells were treated with metHb, ferrylHb, heme (25 or 50 µM, light and dark gray columns, respectively), and recombinant TNF-α (100 ng/mL, black column) for 24 h (*n* = 3/group) to generate cellular inflammatory conditions in an in vitro model of IVH-induced inflammation. In response to metHb, ferrylHb, or heme, significantly elevated IL-8 protein levels were detected already at lower concentrations compared to untreated sample (white bar) as seen after treatment with TNF-α (**A**). In addition, extracellular miRNAs showed a substantial upregulation in the cell culture supernatant of the cells (**B**–**D**). Mean ± SEM are depicted. * *p* < 0.05, ** *p* < 0.01, *** *p* < 0.001, **** *p* < 0.0001 vs. baseline based on ANOVA with Bonferroni’s multiple comparisons test or unpaired t-test. FerrylHb: Ferrylhemoglobin, IL-8: Interleukin-8, IVH: Intraventricular hemorrhage, MetHb: Methemoglobin, miRNA: MicroRNA, TNF-α: Tumor necrosis factor-alpha.

To elucidate the source of miRNAs, in an in vitro model of IVH-induced inflammation, HCPEpiC cells were treated with metHb, ferrylHb, heme (25 or 50 μM), or recombinant TNF-α (100 ng/mL) as control for 24 h to generate cellular inflammatory conditions, followed by miRNA analysis in the cell culture supernatants analyzed by RT-qPCR. Efficacy of pro-inflammatory treatment was monitored via measurement of IL-8 protein levels, while cell death was tested with the detection of lactate dehydrogenase (LDH) activity in cell culture medium. As with TNF-α, there was no substantial elevation in LDH activity induced by Hb metabolites, which excluded the development of significant cell death under these circumstances (data not shown). In response to metHb, ferrylHb, or heme, significantly elevated IL-8 levels were reached already at lower concentrations compared to untreated sample and as seen after treatment with TNF-α (Figure 7A). In addition, extracellular miRNAs showed upregulated levels in the supernatants in the absence of cell death. Interestingly, the oxidative Hb forms and heme resulted in different effects on extracellular miRNA expression of HCPEpiC cells; metHb induced primarily miR-155 and miR-223 expression, while miR-181b was more elevated by ferrylHb and heme at higher concentration (Figure 7B–D). These results suggest that oxidized Hb metabolites and heme induce the activation of NF-κB pathway in HCPEpiC cells with elevated expression and release of pro-inflammatory miRNAs, which may enter into the intraventricular space after IVH.

## 3. Discussion

IVH is a severe complication of prematurity that results in a high neonatal mortality and impaired neurodevelopment among survivors [31]. IVH is accompanied with brain injury because of neuroinflammation that holds a central role in the pathophysiology of IVH [5]. However, the molecular mechanism by which IVH triggers inflammatory response is still under investigation. Extravasation of blood leads to hemolysis with the release of heme and, due to subsequent oxidation of extracellular Hb, oxidative Hb forms accumulate in the intraventricular space [32]. Very recently, elevated metHb, ferrylHb, and heme levels have been demonstrated to elicit intraventricular inflammatory events after the onset of IVH [27]. In parallel, a number of pro-inflammatory chemokines and cytokines are also produced at large quantity in IVH [11,28], while decreased activity of coagulation FVII was found to be an independent risk factor for the development of IVH [12].

miRNAs have been investigated as new pathological mediators and potential biomarkers in various neurological disorders, as reviewed in [24]. Specific miRNAs were found to be involved in neuronal apoptosis after experimental acute cerebral ischemia [25] and altered plasma miRNA levels were associated with the severity of traumatic brain injury [33]. Expression of miRNAs was also profiled in CSF samples in subjects with acute ischemic stroke and let-7c with miR-221 was found to be upregulated in relation to stroke [34]. In SAH, increased miR-21 and miR-221 levels in CSF have been linked to the development of delayed cerebral ischemia [26]. However, the expression of CSF miRNAs after IVH had not been investigated in preterm infants before the present study.

In this study, we selected four pro-inflammatory miRNAs, i.e., miR-155, miR-223, miR-181b, and miR-126, for a quantitative analysis in post-IVH CSF specimens, all of which have been proven to regulate the NF-κB pathway activation [35] and the expression of some particular adhesive receptors on endothelial/epithelial cells, respectively [19,20,21,22,23].

This study has three major findings. First, expression of cell-free miR-223, miR-155, miR-181b, and miR-126 in CSF was elevated in premature infants who suffered from high-grade IVH. IVH-induced upregulation of miRNAs was followed by lowering levels in connection with the elimination of oxidized Hb during the follow-up period. Second, pro-inflammatory IL-8, soluble E-selectin, ICAM-1, and VCAM-1 concentrations were increased in the same CSF samples of IVH, which showed strongly positive correlations with miR-223, miR-155, and miR-181b. Third, augmented miRNA expressions were highly associated with increased levels of oxidized Hb forms and heme after the onset of IVH. All these findings suggest that CSF miR-223, miR-155, and miR-181b levels can indicate pro-inflammatory events as potentially new laboratory biomarkers in IVH preterm infants. The cellular origin of CSF miRNAs may be the affected HCPEpiCs, since hemolysis after IVH as the source could be excluded based on the results of our in vitro experiments.

IVH commonly occurs in premature infants with very low birth weight as a result of germinal matrix hemorrhage [36]. As a consequence of choroid plexus injury and blood–brain barrier dysfunction, CSF protein concentrations rise, causing high total protein and S100B concentrations, while elevated CSF lactate level indicates hypoxia [36]. Substantial inflammatory response induced by Hb metabolites was described in rabbit preterm model of IVH at early phase that was associated with choroid plexus cell death and ultrastructural changes [9]. Poor clinical outcome was connected to increased CSF miR-9 levels in SAH [37], but no data were available regarding which CSF miRNAs are changed in IVH. Hence, in this study, we analyzed the levels of some key pro-inflammatory miRNAs, and miR-223, miR-155, and miR-181b with miR-126 demonstrated elevated expression after the onset of high-grade IVH. Similarly, increased miR-223 levels in CSF were reported in SAH [38,39] and in Alzheimer disease [40]. When samples were split into subgroups based on disease severity, there was no difference in miRNA levels between grades III and IV. On the other hand, there was a time-dependent kinetic of CSF miRNA expression, as lower miRNA levels were determined if CSF samples were drawn only after four weeks of IVH. A downward trend was found by others in CSF miR-92a and let-7b over time in subjects with SAH [41].

Recently, elevated amounts of oxidized Hb forms and heme were detected in CSF specimens of premature IVH subjects [27,42]. Here, we also measured increased levels of metHb, ferrylHb, and heme in these IVH individuals, and a strong association between miRNAs and oxidized Hb metabolites/heme was observed. This relationship was studied by three approaches: (1) Oxidized Hb positive samples showed higher miR-223, miR-155, and miR-181b levels than oxidized Hb negative cases, (2) during the follow-up, there were decreased miRNA levels in those subjects, who became negative for oxidized Hb under treatment, and (3) using Spearman’s test, strong, positive correlation was analyzed between oxidized Hb forms and all the studied miRNAs. These data indicate that oxidized Hb-induced inflammation is strongly linked to the alteration of miRNA expression in CSF. Erdei et al. also described positive correlations between CSF heme and upregulated VCAM-1 and ICAM-1, and IL-8 concentrations in IVH [27]. Here, miRNAs, especially miR-223, showed statistically significant positive correlations with these pro-inflammatory protein biomarkers (Table 2). In addition, soluble E-selectin, ICAM-1, VCAM-1, and IL-8 levels were also augmented in the current IVH vs. non-IVH CSF samples but, surprisingly, they did not vary between grades III and IV. These results indicate that there was no considerable difference in terms of the level of inflammation and cellular activation among these high-grade IVH patients. However, IL-8 level, but not soluble adhesion molecules, was significantly lower in CSF samples obtained at later time points, i.e., over 28 days (Figure 5). In contrast, the levels of adhesion molecules were reduced during the follow-up period with a total elapsed time of 85.4 ± 14.6 days. Substantial decrease in IL-8 and VCAM-1 was shown for up to 60 days of IVH, while ICAM-1 did not change significantly [27]. This type of cytokine and the adhesion molecules above have been considered as sensitive markers of neuroinflammation and cell activation events not only in IVH [27,28] but also in SAH [15] and in bacterial sepsis with meningitis [16]. Based on a recent meta-analysis, chorioamnionitis was described as a risk factor for the development of IVH in very preterm infants [43]. Apart from IL-8, IL-6 level was also elevated in the CSF of infants exposed to chorioamnionitis in association with an increased risk of posthemorrhagic hydrocephalus [44,45]. In our patient cohort, only one preterm infant was exposed to chorioamnionitis and, thus, we could not correlate the expression of CSF miRNAs with this condition.

Besides the characterization of CSF miR-223, miR-155, and miR-181b as novel beneficial biomarkers of preterm IVH, another important implication of this study was to investigate their cellular origin. miRNAs represent a novel way of intercellular communication in CNS with the ability to modulate the function of other cell types under neuronal development and in neurological diseases [24]. As a transport mechanism, miR-9, miR-26a, and miR-124 were detected to be compartmentalized and secreted within extracellular vesicles in CSF after IVH [46]. Moreover, analysis of the source of cell-free miRNAs may lead to a better understanding of their pathophysiological role behind brain injury caused by IVH in preterm infants. Hence, we performed in vitro experiments to address this question on the origin of elevated miRNAs that we measured. Since the correlation analysis showed a positive relationship of miRNA levels with total RBC count (Table 2), one may speculate that increased miRNA levels were merely produced by hemolysis causing a considerable release of intracellular miRNAs from RBCs into the intraventricular space. To address this question, in vitro controlled hemolysis experiments were performed, and miRNA levels were quantified by RT-qPCR in artificially hemolytic vs. nonhemolytic samples; miR-223, miR-155, and miR-181b levels remained unaffected in contrast to RBC-specific miR-16 expression that gradually elevated by increasing degree of RBC contamination [29,30] (Figure 6). Similarly, miR-15b and miR-24 were not modulated by artificial hemolysis either [29]. Accordingly, we excluded the RBC origin of studied pro-inflammatory miRNAs upon in vivo hemolysis after the onset of IVH. Then, using an in vitro model of IVH-induced epithelium inflammation, we stimulated HCPEpiC cells with metHb, ferrylHb, heme, or recombinant TNF-α as control for 24 h for miRNA analysis. In response to metHb, ferrylHb, or heme, extracellular miRNAs showed upregulated levels in the cell culture supernatants. In addition, significantly elevated IL-8 levels were determined, compared to untreated sample, as seen after treatment with TNF-α with no LDH leakage (Figure 7). When we used 100 μM oxidized Hb or heme for the activation of the cells, miRNA expressions were still increased, but in the presence of LDH leakage, showing 2–3-fold higher enzyme activity than control samples (data not shown). These results suggest that oxidized Hb metabolites and heme induced the activation of the NF-κB pathway in these HCPEpiCs with the active release of pro-inflammatory miRNAs, which may enter the intraventricular space upon IVH. These data are in accordance with former publications about the pro-inflammatory actions of heme and other oxidized Hb-derived forms on ECs [10,47]. For instance, heme triggered von Willebrand factor and P-selectin surface exposure on EC via TLR4 activation and NF-κB signaling [10], while others reported that ferrylHb induced E-selectin, VCAM-1, and ICAM-1 expression on vascular EC through the activation of transcription factors via NF-kB pathway [48].

This study has some limitations that will be addressed in a subsequent work. First, a larger sample size needs to be investigated to validate these CSF miRNAs with their functional role in IVH. Second, other miRNAs, present rich in brain, could be parallelly evaluated to analyze their diagnostic accuracy. Third, the levels of these studied miRNAs need to be further quantified to explain their clinical relevance in terms of functional outcomes at two years of age as an important endpoint following treatment for IVH [49].

In conclusion, this study demonstrates that IVH induces upregulation of pro-inflammatory miR-223, miR-155, and miR-181b in CSF in correlation with oxidized Hb forms and protein biomarkers E-selectin, ICAM-1, and VCAM-1. Overall, induced CSF miRNAs effectively monitor inflammatory conditions as potential biomarkers in preterm IVH.

## 4. Materials and Methods

### 4.1. Study Participants

In this study, 47 preterm infants (21 females and 26 males) were involved after diagnosis with grade III (*n* = 21) or grade IV IVH (*n* = 26) with a mean gestational age at birth of 28.2 ± 3.2 weeks and birth weight of 1235 ± 610 g (Table 1). As clinical controls, we recruited 14 non-IVH preterm infants (7 females and 7 males) with a mean gestational age of 32.0 ± 6.8 weeks and birth weight of 2046 ± 1281 g at delivery who were diagnosed with congenital hydrocephalus without bleeding or were investigated via lumbar puncture for routine meningitis/infection evaluation and were then verified as sterile and, thus, meningitis or severe infection was excluded (Table 1). Cranial ultrasound examination was performed in combination with laboratory analyses to confirm the presence or absence of IVH, and the severity of IVH was determined by the clinicians (Department of Pediatrics, University of Debrecen). This study was approved by the Scientific and Research Ethics Committee of the University of Debrecen (permit number: 4876-2017) in accordance with the Declaration of Helsinki. Parental consent forms were signed by the parents of the infants involved in this study.

### 4.2. CSF Sample Collection and Preparation

CSF samples (*n* = 47) were collected by spinal tap or ventricular reservoir puncture at the Department of Neurosurgery, University of Debrecen, at 23.8 ± 10.7 days after the onset of IVH. In the case of 18 subjects, additional CSF samples were available during the follow-up period with a total elapsed time of 85.4 ± 14.6 days. In parallel, 14 CSF specimens were obtained from clinical controls. In this study, the leftover CSF specimens (*n* = 79) obtained for diagnostic purposes were examined. No CSF was obtained exclusively for inclusion into this study. Within 30 min after collection, CSF samples were centrifuged at 650 g for 5 min at 4 °C, and cell-free supernatants were immediately frozen at −80°C until analysis.

### 4.3. Human Choroid Plexus Epithelial Cell Culture

Human primary choroid plexus epithelial cells (HCPEpiC, ScienCell™ Research Laboratories, Carlsbad, CA, USA) were cultured in Epithelial Cell Medium (ScienCell) containing 2% fetal bovine serum (FBS, ScienCell), 1% Epithelial Cell Growth Supplement (ScienCell), and 1% Penicillin/Streptomycin solution (ScienCell) at 37 °C and 5% CO_2_. For culturing these cells, the manufacturer’s protocol was followed. Cell density was set to 6000 cells per cm^2^ and poly-L-lysine (ScienCell) -coated (2 µg/cm^2^) BioLite cell culture flasks (Thermo Scientific, Rochester, NY, USA) were used.

### 4.4. Treatment of Human Choroid Plexus Epithelial Cells with Oxidative Hb Forms and Heme

HCPEpiCs (2 × 10^5^/well) were then treated in BioLite 6-well plates (Thermo Scientific, Rochester, NY, USA) with metHb, ferrylHb, heme (25–50 µM), or recombinant TNF-α (100 ng/mL, Gibco, Carlsbad, CA, USA) as control for 24 h to replicate IVH-induced inflammatory cellular conditions in vitro. MetHb and ferrylHb were prepared from fresh peripheral blood samples obtained from healthy volunteers, as we have previously described [27]. After treatment, supernatants were collected and stored at −80 °C before RNA isolation and measurement of protein biomarkers. To analyze the degree of cell death caused by treatments, LDH leakage was determined in the supernatants of stimulated HCPEpiC cell cultures via photometric measurement of catalytic LDH activity on a Cobas^®^ 6000 analyzer (Roche Diagnostics, Mannheim, Germany).

### 4.5. Extraction of miRNAs from CSF Samples and HCPEpiC Supernatants

Thawed CSF samples were first centrifuged at 10,000 g for 1 min and 200 µL of cell-free supernatants were spiked-in with 10 pmol mirVana^™^ cel-miR-39 mimic (Ambion, Austin, TX, USA, ID:MC10956). For extracellular miRNA extraction, miRNeasy Kit (Qiagen, Hilden, Germany) was used according to the manufacturer’s recommendations. In parallel, supernatants of activated HCPEpiCs were prepared in the same conditions as above, but 225 µL of supernatants were spiked-in with 5 pmol mirVana^™^ cel-miR-39 mimic. For the extraction of extracellular miRNAs, NucleoSpin^®^ miRNA Kit (Macherey-Nagel, Düren, Germany) was used according to the manufacturer’s recommendations. The purity and concentration of isolated RNA samples were verified by a NanoDrop^™^ spectrophotometer (Thermo Scientific, Wilmington, DE, USA), and samples were stored at −80 °C.

### 4.6. miRNA-Specific Stem-Loop RT-qPCR Analysis

The expressions of cell-free miRNAs (i.e., miR-223, miR-155, miR-181b, and miR-126) were quantified by miRNA-specific Universal ProbeLibrary (UPL) probe-based stem-loop RT-qPCR method as we previously described [23,50]. Briefly, this quantification technique included two steps: (1) miRNAs were transcribed into cDNA via reverse transcription using miRNA-specific stem-loop RT primer (500 nM, Integrated DNA Technologies, Leuven, Belgium) and TaqMan^™^ MicroRNA Reverse Transcription Kit (Applied Biosystems, Foster City, CA, USA) and (2) miRNA quantification was performed by RT-qPCR using designed universal reverse primer (100 μM, Sigma-Aldrich, St. Louis, MO, USA), miRNA-specific forward primer (100 μM, Integrated DNA Technologies), and UPL probe #21 (10 μM, Roche Diagnostics) with recombinant Taq DNA polymerase (5 U/μL, Thermo Scientific, Vilnius, Lithuania) and dNTPs (2.5 mM, Thermo Fisher Scientific). All the measurements were run in triplicate on a QuantStudio^™^ 12K Flex qPCR instrument (Applied Biosystems). For normalization, the exogenous ‘spike-in’ control cel-miR-39 was measured in all the samples with the same method as above. Primers and qPCR assays were designed by the software developed by Czimmerer et al. [51], and oligonucleotides that were used in this study are listed in Supplementary Table S1.

### 4.7. Determination of Hemoglobin Forms and Heme Levels in CSF Samples

The absorbance spectra (250–700 nm) of CSF samples were measured with a spectrophotometer (NanoDrop^™^, Thermo Scientific), as we recently performed [27]. Briefly, concentrations of metHb and ferrylHb were calculated from the absorbance values measured at 541, 576, and 630 nm, using the absorption coefficients and equations determined by others [52]. Total heme concentration of CSF samples was determined by using a QuantiChrom^™^ Heme Assay Kit (Gentaur Ltd., London, UK) according to the manufacturer’s instructions.

### 4.8. Measurement of Soluble Adhesion Molecule Concentrations

To perform ELISA, CSF samples and HCPEpiC supernatants were first centrifuged at 10,000 g for 1 min at room temperature (RT). Soluble E-selectin, VCAM-1, ICAM-1, and IL-8 protein concentrations were measured by commercially available ELISA kits based on the manufacturer’s protocol (R&D Systems, Minneapolis, MN, USA).

### 4.9. Other Laboratory Analyses

Routine laboratory parameters were performed at the Department of Laboratory Medicine, University of Debrecen, for diagnostic purposes. Serum C-reactive protein (CRP) and PCT levels were measured by an electro-chemiluminescent immunoassay on a Cobas^®^ e411 analyzer (Roche Diagnostics). Whole blood WBC and platelet count as well as Hb concentration were determined by an Advia^®^ 2120 Hematology System analyzer (Bayer Diagnostics, Tarrytown, NJ, USA). In CSF samples, RBC and WBC counts were analyzed on a Sysmex^®^ XN-1000^™^ hematology analyzer (Sysmex, Kobe, Japan), while CSF total protein level was determined by immunoturbidimetry and CSF lactate concentration was analyzed by a colorimetric test on a Cobas^®^ 6000 analyzer (Roche Diagnostics). S100B level in CSF was measured by a chemiluminescence immunoassay (Liaison XL^™^, DiaSorin, Saluggia, Italy).

### 4.10. In Vitro Controlled Hemolysis Experiments

In vitro controlled hemolysis experiments were performed to evaluate the impact of hemolysis on these studied circulating miRNAs based on a former publication [30]. For this purpose, due to lacking additional CSF samples of study participants, peripheral blood samples were collected into Vacutainer^®^ tubes containing K_2_-EDTA (Becton Dickinson, San Jose, CA USA) from healthy volunteers (3 females and 2 males) who underwent a detailed medical anamnesis, physical examination, and routine laboratory tests and were free of any acute cardiovascular, inflammatory, or metabolic disease or cancer. Within 30 min of blood collection, RBC and plasma samples were separated by multiple centrifugation steps: (1) Tubes were centrifuged at 200 g for 10 min at RT, (2) the upper layer of plasma was carefully transferred into clean tubes and was centrifuged again at 1500 g for 15 min at RT to gain cell-free plasma, and (3) the residual plasma and buffy coat were removed by pipetting, and RBC suspensions were obtained from the bottom of tubes. Hemolysis was artificially mimicked in the plasma by adding RBCs in different ratios (0, 1, 2.5, 5, 10, and 50% *v/v*) into six different samples (Supplementary Figure S3A,B). After a vigorous mixing by vortex, samples (250 µL) were spiked-in with the synthetic cel-miR-39 (10 pmol), lysed with 750 µL TRI Reagent^®^ LS (Sigma-Aldrich), and total RNA was isolated according to the manufacturer’s instructions. The miRNA content of hemolytic and uncontaminated control samples was quantified by the same method as above. As a positive control, RBC-specific miR-16 [29] was used to monitor the efficacy of induced hemolysis. Sequences of primers for this miRNA analysis are listed in Supplementary Table S1.

### 4.11. Statistical Analysis

Kolmogorov–Smirnov test was used for evaluation of the normality of data. Results are expressed as mean ± SD or SEM and median with (interquartile range), as appropriate. To compare the data of two groups, we applied unpaired or paired t-test, Mann–Whitney U test, or Wilcoxon matched-pairs signed rank test and chi-squared test. Comparison of multiple groups was performed using ANOVA, Kruskal–Wallis test, or Friedman test with multiple comparisons tests, as appropriate. Correlations between miRNA levels and other parameters were determined using Spearman’s test. A strong positive correlation was defined as a value of Spearman’s correlation coefficient (*r*) > 0.4. Statistical significance was defined when *p* value was < 0.05. Statistical analyses were performed using GraphPad Prism software (version 6.01, La Jolla, CA, USA).

## Figures and Tables

**Table 1:** Baseline demographical and laboratory parameters of preterm IVH and control patients (n indicates the number of involved patients). Data are expressed in mean ± SD or median with (interquartile range) as appropriate. For statistical analysis, chi-square test and unpaired t-test or Mann–Whitney U test were used as appropriate.

Characteristics	IVH Patients (*n* = 47)	Clinical Controls (*n* = 14)	*p* Value
Gestational age (weeks)	28.2 ± 3.2	32.0 ± 6.8	0.3427
Gender (male/female)	26/21	7/7	0.7259
Birth weight (g)	1235 ± 610	2046 ± 1281	0.240
Severity of IVH (grade III/IV)	21/26		
Whole blood WBC count (G/L)	12.0 (9.77–13.80)	10.26 (8.64–12.33)	0.0940
Whole blood Hb (g/L)	120 (102–135)	107 (99–130)	0.4474
Whole blood platelet count (G/L)	384 (286–484)	370 (288–509)	0.963
Serum CRP (mg/L)	1.34 (0.60–3.76)	0.50 (0.50–4.51)	0.2192
Serum PCT (µg/L)	0.29 (0.19–0.50)	0.15 (0.10–0.19)	0.0066
CSF RBC count (cells/µL)	4267 (1536–10400)	2 (1–518)	<0.0001
CSF WBC count (cells/µL)	52 (23–207)	8 (4–26)	0.0005
CSF total protein (mg/L)	1799 (1376–2498)	758 (448–1510)	0.0007
CSF S100B (µg/L)	7.49 (5.01–10.40)	3.23 (1.12–5.46)	0.0003
CSF lactate (mmol/L)	3.47 (2.58–3.70)	2.16 (1.55–2.45)	0.0002
CSF oxidized Hb (µmol/L)	36.98 ± 54.64	1.82 ± 5.84	0.0005
CSF total heme (µmol/L)	226.3 ± 262.9	0.78 ± 2.81	<0.0001

CSF: Cerebrospinal fluid, CRP: C-reactive protein, Hb: Hemoglobin, IVH: Intraventricular hemorrhage, PCT: Procalcitonin, RBC: Red blood cell, S100B: S100 calcium-binding protein B, WBC: White blood cell.

**Table 2:** Correlation analysis of CSF miRNA expression with pro-inflammatory protein biomarkers and oxidized Hb forms in IVH. Results are expressed as *r* (correlation coefficient) and *p* values according to the comparison between respective corresponding parameters. Strong statistically significant positive correlations are highlighted in bold. The relationship between miRNA levels and other parameters was characterized using Spearman’s test.

	CSF RBC	CSF WBC	E-selectin	ICAM-1	VCAM-1	IL-8	Ox. Hb	Total Heme
miR-223	***r* = 0.5293** ***p* < 0.0001**	***r* = 0.5135** ***p* < 0.0001**	***r* = 0.4244** ***p* = 0.0002**	*r* = 0.3683 *p* = 0.0009	***r* = 0.5101** ***p* < 0.0001**	***r* = 0.5049** ***p* < 0.0001**	***r* = 0.5931** ***p* < 0.0001**	***r* = 0.6281** ***p* < 0.0001**
miR-155	***r* = 0.4241** ***p* < 0.0001**	*r* = 0.2874 *p* = 0.0107	*r* = 0.2670 *p* = 0.0110	*r* = 0.2721 *p* = 0.0070	*r* = 0.3733 *p* = 0.0036	*r* = 0.3160 *p* = 0.0097	***r* = 0.4735** ***p* < 0.0001**	***r* = 0.5394** ***p* < 0.0001**
miR-181b	*r* = 0.3554 *p* = 0.0014	*r* = 0.2254 *p* = 0.0487	*r* = 0.2680 *p* = 0.0111	*r* = 0.2397 *p* = 0.0187	*r* = 0.2994 *p* = 0.0212	*r* = 0.3486 *p* = 0.0041	***r* = 0.4198** ***p* = 0.0001**	***r* = 0.4510** ***p* < 0.0001**
miR-126	*r* = 0.2975 *p* = 0.0057	*r* = 0.0865 *p* = 0.4933	*r* = 0.3588 *p* = 0.0014	*r* = 0.2133 *p* = 0.0514	*r* = 0.1832 *p* = 0.2030	*r* = 0.3303 *p* = 0.0129	*r* = 0.2432 *p* = 0.0323	*r* = 0.3558 *p* = 0.0010

CSF: Cerebrospinal fluid, ICAM-1: Intercellular adhesion molecule 1, IL-8: Interleukin-8, miRNA: MicroRNA, Ox. Hb: Oxidized hemoglobin, RBC: Red blood cell, VCAM-1: Vascular cell adhesion molecule 1, WBC: White blood cell.

## Supplementary Materials

Supplementary materials can be found at https://www.mdpi.com/1422-0067/21/18/6870/s1. Figure S1. Routine laboratory parameters of grade III (*n* = 21) and grade IV (*n* = 26) IVH patients as well as clinical control subjects (*n* = 14). Figure S2. Reduced E-selectin (A), ICAM-1 (B), and VCAM-1 (C) concentrations were analyzed in the follow-up (oxidized Hb negative) CSF samples in comparison to baseline specimens containing oxidized Hb forms (*n* = 11). Figure S3. Graphical representation of in vitro controlled hemolysis experiments. Table S1. Sequences of primers for the analysis of cell-free mature miRNAs.

## Abbreviations

CSF	cerebrospinal fluid
CNS	central nervous system
CRP	C-reactive protein
DCI	delayed cerebral ischemia
EC	endothelial cell
ELISA	enzyme-linked immunosorbent assay
Hb	hemoglobin
HCPEpiC	human choroid plexus epithelial cell
ICAM-1	intercellular adhesion molecule 1
IL	interleukin
IVH	intraventricular hemorrhage
LDH	lactate dehydrogenase
miRNA	microRNA
NF-κB	nuclear factor kappa-B
Ox. Hb	oxidized hemoglobin
PCT	procalcitonin
RBC	red blood cell
RT	room temperature
RT-qPCR	real-time quantitative polymerase chain reaction
S100B	S100 calcium-binding protein B
SAH	subarachnoid hemorrhage
TLR4	Toll-like receptor 4
TNF-α	tumor necrosis factor alpha
UPL	Universal ProbeLibrary
VCAM-1	vascular cell adhesion molecule 1
WBC	white blood cell
