# Supplementary material for: Elevated Pro-Inflammatory Cell-Free MicroRNA Levels in Cerebrospinal Fluid of Premature Infants after Intraventricular Hemorrhage
Source: Int J Mol Sci. 2020 Sep 19;21(18):6870. doi: 10.3390/ijms21186870 (PMC7557369; doi:10.3390/ijms21186870)
Supplement: Supplementary file 1 [file ijms-21-06870-s001.pdf]

Supplementary materials

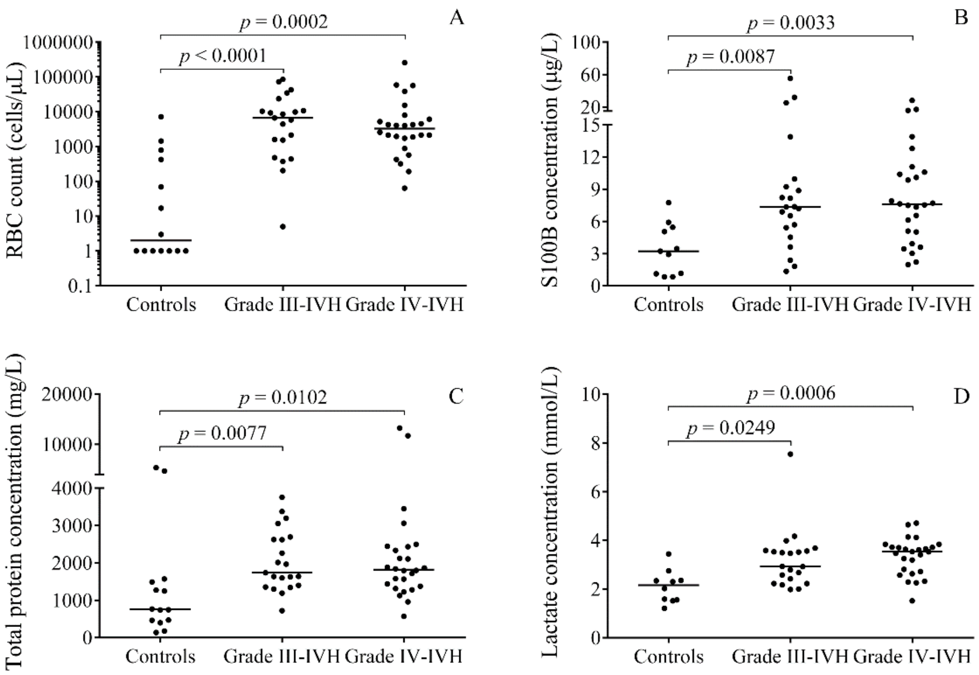

Supplementary Figure 1. Routine laboratory parameters of grade III ($n = 21$) and grade IV ($n = 26$) IVH patients as well as clinical control subjects ($n = 14$). In baseline cerebrospinal fluid samples, RBC count (**A**) and the levels of S100B (**B**), total protein (**C**), and lactate (**D**) were significantly higher in both IVH severity groups versus controls; however, there was no difference between the two subcohorts. For comparison, Kruskal-Wallis test with Dunn's multiple comparisons test was performed. IVH: intraventricular hemorrhage, RBC: red blood cell, S100B: S100 calcium-binding protein B.

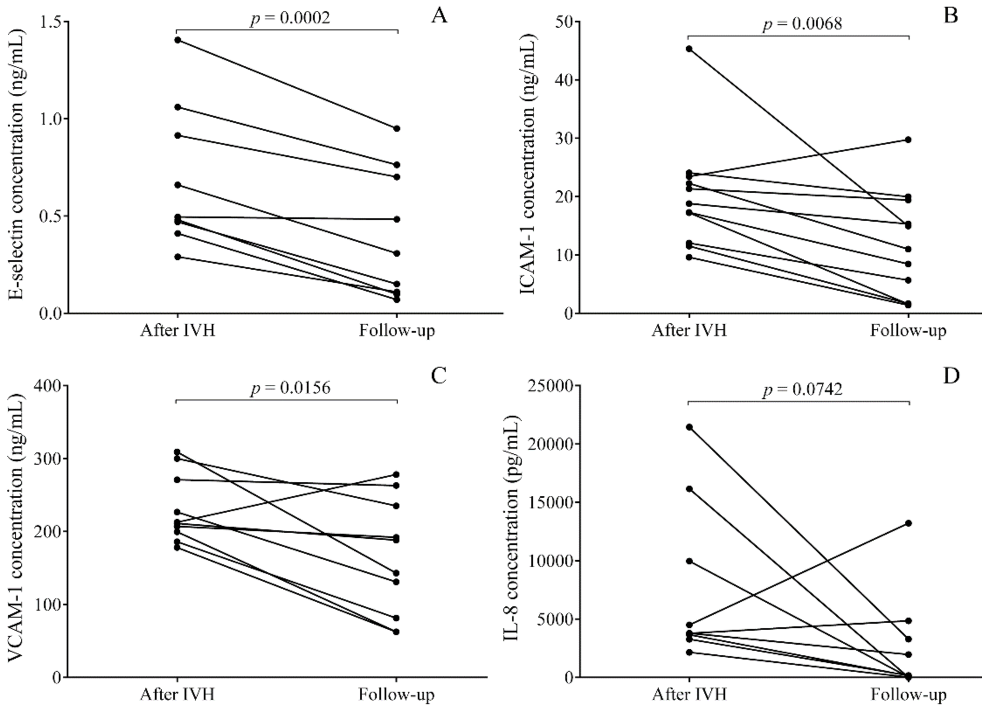

Supplementary Figure 2. Reduced E-selectin (**A**), ICAM-1 (**B**), and VCAM-1 (**C**) concentrations were analyzed in the follow-up (oxidized Hb negative) CSF samples in comparison to baseline specimens containing oxidized Hb forms ($n = 11$). In contrast, IL-8 (**D**) did not show a significant alteration over time (9 samples were available for this analysis). Dots represent single values of the pairs. Wilcoxon matched-pairs sign rank test or paired t-test were performed for the comparisons. CSF: cerebrospinal fluid, Hb: hemoglobin, ICAM-1: intercellular adhesion molecule 1, IL-8: interleukin-8, IVH: intraventricular hemorrhage, VCAM-1: vascular cell adhesion molecule 1.

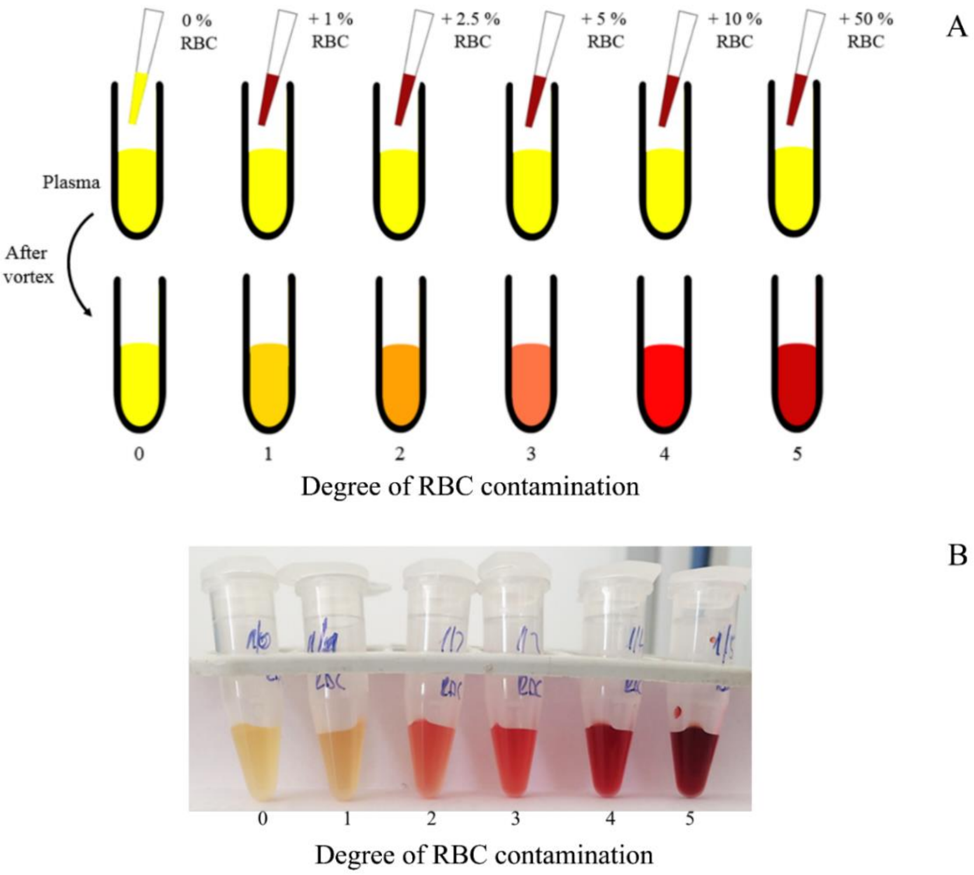

Supplementary Figure 3. Graphical representation of in vitro controlled hemolysis experiments. Top panel (**A**) illustrates the performance of RBC contamination (0-50 % v/v) of plasma samples (n=5/condition), while the bottom part of the figure (**B**) depicts the colorimetric scale of induced hemolysis (tube 0 means the non-hemolytic control sample, and tube 5 contains the highest amount of RBC). RBC: red blood cell.

Supplementary Table 1. Sequences of primers for the analysis of cell-free mature miRNAs. In case of stem-loop reverse transcription primers, the miRNA specific sequences are highlighted in red. Universal ProbeLibrary probe #21 (5′ – TGGCTCTG – 3′) was used for the RT-qPCR measurements. miRNA: microRNA, RT-qPCR: real-time quantitative polymerase chain reaction.

miRNAs	Stem-loop primers for reverse transcription (5′ – 3′)	Forward primers (5′ – 3′)	Universal reverse primer (5′ – 3′)
miR-223-3p	GTTGGCTCTGGTGCAGGGTCCGAGG TATTCGCACCAGAGCCAAC TGGGGT	GTTGGGTGTCAGTTTGTC AAAT	GTGCAGGGTCCGAGGT
miR-155-5p	GTTGGCTCTGGTGCAGGGTCCGAGG TATTCGCACCAGAGCCAAC ACCCCT	GTGGGTTAATGCTAATCG TGAT	GTGCAGGGTCCGAGGT
miR-181b-5p	GTTGGCTCTGGTGCAGGGTCCGAGG TATTCGCACCAGAGCCAAC ACCCAC	GTTTGAACATTCATTGCT GTCG	GTGCAGGGTCCGAGGT
miR-126-3p	GTTGGCTCTGGTGCAGGGTCCGAGG TATTCGCACCAGAGCCAAC CGCATT	GGGTCGTACCGTGAGTAAT	GTGCAGGGTCCGAGGT
miR-16-5p	GTTGGCTCTGGTGCAGGGTCCGAGG TATTCGCACCAGAGCCAAC CGCCAA	GTTTGGTAGCAGCACGTA AATA	GTGCAGGGTCCGAGGT
cel-miR-39-3p	GTTGGCTCTGGTGCAGGGTCCGAGG TATTCGCACCAGAGCCAAC CAAGCT	GTGTCACCGGGTGTAAATC	GTGCAGGGTCCGAGGT
